# Cumulative Lifetime Violence, Gender Role Conflict, and Cardiovascular Disease Risk in Eastern Canadian Men

**DOI:** 10.1177/15579883231176996

**Published:** 2023-06-07

**Authors:** Kelly Scott-Storey, Sue O’Donnell, Charlene D. Vincent, Jeannie Malcolm, Judith Wuest

**Affiliations:** 1 Faculty of Nursing, University of New Brunswick, Fredericton, NB, Canada

**Keywords:** cumulative lifetime violence, cardiovascular risk, Framingham 30-year risk, gender role conflict, men, trauma- and violence-informed care

## Abstract

Despite violence being a chronic stressor that negatively affects health through allostatic overload and potentially harmful coping behaviors, the relationship between cumulative lifetime violence severity (CLVS) and cardiovascular disease (CVD) risk in men has received little attention and the role of gender has not been considered. Using survey and health assessment data from a community sample of 177 of eastern Canadian men with CLVS as target and/or perpetrator, we developed a profile of CVD risk measured by the Framingham 30-year risk score. We tested the hypothesis that CLVS measured by the CLVS-44 scale has direct and specific indirect effects through gender role conflict (GRC) on 30-year CVD risk using parallel multiple mediation analysis. Overall, the full sample had 30-year risk scores 1.5 times higher than their age-based Framingham reference normal risk scores. Men classified as having *elevated* 30-year CVD risk (*n* = 77) had risk scores 1.7 times higher than reference normal. Although the direct effects of CLVS on 30-year CVD risk were not significant, indirect effects of CLVS through GRC, specifically Restrictive Affectionate Behavior Between Men, were significant. These novel results reinforce the critical role of chronic toxic stress, particularly from CLVS but also from GRC, in influencing CVD risk. Our findings highlight the need for providers to consider CLVS and GRC as potential antecedents to CVD and to routinely use trauma- and violence-informed approaches in the care of men.

Globally, coronary heart disease and stroke remain the leading causes of disease burden despite improved understanding of cardiovascular disease (CVD) risk factors and advancements in medical care and rehabilitation (Kivimäki & Steptoe, 2018). Scott-Storey (2013) positioned lifetime abuse/violence as a chronic *stressor* affecting development of CVD in two ways: (a) directly through allostatic overload that causes long-term physiological changes in the metabolic, neuroendocrine, hemostatic, immune, and cardiovascular systems and (b) indirectly through behaviors (e.g., smoking, overeating) initiated or escalated to manage abuse-related distress. From a review of meta-analyses published in the past decade, Kivimäki and Steptoe (2018) concluded that, in general populations, extreme chronic stress in childhood from trauma experiences (e.g., physical abuse, sexual abuse, domestic violence, household substance abuse) known as toxic stress doubled the risk for CVD in adulthood, an effect comparable to that of smoking or diabetes (Kivimäki & Steptoe, 2018).

Despite violence being a pervasive public health problem negatively affecting men’s morbidity and mortality globally (Haegerich & Hall, 2011), the relationship between violence and CVD in men has not been comprehensively examined. Specifically, the cumulative effect of experiences of violence as perpetrator and target in childhood through adulthood on CVD risk has been neglected. Few studies of violence and CVD risk disaggregate findings by sex, and the role of gender rarely is considered. How men experience and respond to violence is influenced by gender role socialization (Fallot & Bebout, 2012; Scott-Storey et al., 2022). Internalized socialized masculine roles become standards for men’s thoughts, beliefs, and behaviors (Connell, 2005). Gender role conflict (GRC) experienced when such standards are incompatible with situational demands leads to fear, avoidance, and discomfort (O’Neil, 2008). For men with violence histories, GRC may be an additional stressor that increases vulnerability for CVD. Our current exploration of the relationship between trauma in the form of cumulative lifetime violence (CLV), gender and CVD risk in men draws on existing conceptual and empirical knowledge. Our analysis is important because many primary care providers do not recognize chronic and toxic violence-related stress and GRC as possible antecedents to chronic health problems such as CVD; nor are vital trauma- and violence-informed approaches routinely used in care of men (Hamberger et al., 2019).

## Background

Studies of associations between violence and CVD have largely focused on women and most studies that include men and women report only aggregated findings. Overall, a consistent positive association between self-reports of abuse/violence and neglect in childhood and CVD in adulthood was reported in a systematic review of multiple cross-sectional studies (Suglia et al., 2015). Secondary analysis of U.K. biobank data indicated that documented childhood abuse and neglect was related to increased CVD risk; in men, hypertensive disease was related to physical and emotional maltreatment, but not sexual abuse, and cerebrovascular disease was associated with physical neglect and emotional abuse and neglect (Soares et al., 2020). In a retrospective study of a large (>80,000) U.K. primary care sample of adults (41.7% male), researchers discerned that those with confirmed child maltreatment had significantly higher incidence for ischemic heart disease (1.6), stroke/transient ischemic attack (2.2), and hypertension (1.4) than matched counterparts without this history (Chandan et al., 2020).

Sexual abuse at any point in the lifespan has been associated with CVD in adulthood. Survey data from 10 countries illustrated that men and women with experiences of childhood sexual abuse compared with those without were 4 times more likely to self-report heart disease (Scott et al., 2011). National U.S. surveillance data identified that the odds for myocardial infarction for boys who experienced forced penetrative sex by someone at least 5 years older were 3 times higher than for those who had not (Fuller-Thomson et al., 2012). A meta-analysis of studies of CVD and sexual violence/abuse at any point in the lifetime (77.1% women) established that the odds of CVD in adulthood were 1.3 for those exposed to sexual violence compared with those not exposed (Jakubowski et al., 2021). Effects of sexual abuse as child only or as both a child and an adult were larger for CVD risk in adulthood than effects of sexual abuse experienced only as an adult; this suggests that childhood experiences may be uniquely influential. No studies of violence perpetration in childhood and CVD in adulthood were found.

Studies of CVD and violence experienced in adulthood have focused mostly on intimate partner violence (IPV) among samples of women (Suglia et al., 2015). Analysis of U.S. risk factor survey data determined an odds ratio (OR) of 1.4 for stroke in men who reported lifetime IPV as a target (i.e., victim; Breiding et al., 2008). U.S. longitudinal youth survey data revealed that severe IPV as both target and perpetrator between ages 15 and 22 years was associated with incident hypertension as adult men, but neither perpetration nor victimization alone was significant (Clark et al., 2014). A second analysis identified that men and women with experience as *both* IPV target and perpetrator, but *not* one or the other, were more likely to have increased CVD risk measured by the Framingham 30-year risk score (Clark et al., 2016). Workplace violence has also been implicated in greater CVD risk. In a multi-cohort Scandinavian sample of men and women over an average of 12.4 years, Xu et al. (2019) reported a significantly greater risk for CVD to be associated with past year workplace bullying (1.6) and workplace violence (1.3; Xu et al., 2019). This empirical evidence supports the relationships between lifetime violence, particularly in childhood, and CVD in adult men.

However, these findings are limited by narrow conceptualizations and measures of violence and research designs that disregard the co-occurring and interconnecting experiences of violence as target and/or perpetrator across the lifespan (see Hamby & Grych, 2013; Scott-Storey, 2011). Deleterious health effects are attributed to one or two types of violence (e.g., physical or sexual) occurring in one period of the life course (i.e., childhood or adulthood) neglecting the cumulative impact of diverse violence experiences across the lifespan. In the study of CVD, investigators have noted the need for multi-dimensional measures that capture the chronicity and severity of contact and non-contact violence across the lifespan, as targets and/or perpetrators (Jakubowski et al., 2021; Suglia et al., 2015). Scott-Storey et al. (2020) addressed these concerns through the development of a comprehensive tool, the 44-item Cumulative Lifetime Violence Severity (CLVS) scale for men that captures dimensions of severity including timing in the lifespan (childhood, adulthood), role (target, perpetrator), type (physical, psychological, sexual), context (family, peer, intimate partner, workplace, community), frequency, and degree of distress. Another weakness is measurement of CVD commonly by self-report of a diagnosis of heart disease or hypertension (Suglia et al., 2015). A more objective indicator is the 30-year Framingham Risk score which quantifies CVD risk over 30 years for people ages 20 to 59 based on readily measured risk factors of sex, age, systolic blood pressure (BP), anti-hypertensive treatment, smoking, diabetes mellitus, total cholesterol (TC), and high-density lipoprotein (HDL) cholesterol (Pencina et al., 2009).

Credibility of current research findings about violence and CVD in men is also threatened by the limited consideration of masculine gender, that is understanding and expression of what it means to be a man. Gender is a social construct affecting roles, responsibilities, occupations, and behaviors associated with CVD risk as well as amenability to prevention and treatment (Tadiri et al., 2021). Some studies support intersections between gender and other social determinants of health (SDOH) that can affect the development and course of CVD. Smith and Dumas (2019) observed that masculine beliefs about autonomy in how men should care for their bodies and manage disease differed by income level and influenced cardiac rehabilitation. More specifically, lower income men perceived guidelines to modify lifestyle as a threat to autonomy and economic security, downplayed CVD severity and continued to work whereas middle-income men viewed guideline adherence as enhancing autonomy through improved health and maintenance of economic position over time. Uzogara (2019) observed education and masculine beliefs in Black men intersected as predictors of CVD health (body mass index, systolic BP). Adherence to masculine standards of displaying toughness was linked to poorer CVD health in college-educated men and better CVD health in less educated men who experienced less stress from isolation and discrimination for their *tough* behavior than those better educated. Others have examined CVD risk according to gender-related characteristics; in a large population study, Bolijn et al. (2021) identified that primary earner status, a gender role commonly assigned to men, was associated with a higher CVD risk estimated by Systematic Coronary Risk Evaluation for both men and women. We found no studies examining GRC and CVD risk among men including those with histories of violence. Violent behavior and victimization in men have been associated with GRC (O’Neil, 2008). We propose that stress from conflict between the demands of traditional masculine socialization and demands of everyday situations may mediate the relationship between CLV and CVD risk.

Knowledge of health behaviors and SDOH relevant to CVD among men with histories of CLV is also scant. Although coping behaviors such as substance use (tobacco, alcohol cannabis), poor dietary habits, and physical inactivity (Kreatsoulas et al., 2018; Scott-Storey, 2013) used to manage violence-related stress are also well-known modifiable risk factors for CVD (Joseph et al., 2017), intersections among these behaviors, CVD and CLV have not been studied. For example, frequent cannabis use among men was associated with higher CLVS (O'Donnell et al., 2020) and, also, with atherosclerotic CVD (Skipina et al., 2022) but only in separate studies. Similarly, despite unemployment among men being associated with experiencing and witnessing abuse in childhood (Liu et al., 2013) and unemployment, and low income being consistently linked to more severe CVD (Lennon et al., 2018; Schultz et al., 2018), violence history is commonly ignored in the examination of SDOH and CVD in men.

Beyond health behavior and SDOH, biophysical CVD risk factors are critical indictors of CVD risk. The absence of CVD risk factors at 50 years of age has been related to low lifetime CVD risk (Lloyd-Jones et al., 2006). Using a risk factor assessment tool such as the Framingham 30-year risk score to identify those men most vulnerable at an early age provides intervention opportunities for behavioral or social change. Findings may inform the development and implementation of nuanced health promotion strategies using trauma- and violence-informed (TVI) approaches that confirm and address the impact of past and ongoing violence, dominant gender norms, and SDOH on men’s cardiac health and risk behavior (Wathen et al., 2021).

Our aim is to expand knowledge of CVD risk among men with CLV in a community sample of eastern Canadian men who took part in the exploratory *Men’s Violence, Gender, and Health Study (MVGHS).* Using data from both an online survey and an in-person CVD health assessment, our purpose was to develop a profile of CVD risk among men with CLV experience as target and/or perpetrator. Specific goals were to: (a) classify Framingham 30-year lipid hard CVD (hereafter called *30-year CVD*) risk scores by Framingham 30-year CVD risk groups, compare 30-year CVD risk scores to normal risk scores, overall and by 30-year CVD risk groups, and compare known CVD risk factors by 30-year CVD risk groups; (b) develop a descriptive comparative profile of CLVS, GRC, SDOH, and health behaviors by 30-year CVD risk groups; and (c) test the hypothesis that CLVS has direct and specific indirect effects through GRC on 30-year CVD risk scores.

## Method

The *MVGHS* online survey was completed by a community convenience sample of 685 individuals who identified as men and met the inclusion criteria of being English-speaking, age 19 to 65 years, a resident of eastern Canada and willing to take part in a study of violence, gender, and health. Following ethical approval from the University of New Brunswick Research Ethics Board (REB#2014-035), men were recruited using community contacts and online classified advertisements. Respondents were emailed a Letter of Information and an online link for eligibility and consent. Those who gave online consent proceeded to the online survey. Upon survey completion, all participants were directed to a debriefing page with strategies and resources to manage any subsequent distress. Each man received an honorarium of 20 Canadian dollars (CAD). A subsample of 244 men living in the province of New Brunswick (NB) who consented to an in-person CVD health assessment are the focus of the current analysis. CVD assessments were completed between April 2016 and December 2017 by Registered Nurses and senior nursing students in health facilities, workplaces, libraries, or universities. Assessments were interpreted according to accepted guidelines (see Table 1) and participants were given written results that were reviewed and used as a basis for CVD-related health teaching. Those with health check results that fell outside of acceptable parameters received recommendations for follow-up with a health provider. At the end of the assessment, men were given a written copy of the survey debriefing page, and a 20 CAD honorarium, and offered reimbursement for travel.

**Table 1.** Classification of Biophysical Measures

Blood pressure^a^	Systolic	Diastolic
Normal	<120	<80
Prehypertension/high normal	120–139	80–89
High	>140	>90
Hypertensive	≥160	≥100
Total blood cholesterol^b^		
Desirable	Borderline high	High
<5.2 mmol/L	5.2–6.2 mmol/L	>6.2 mmol/L
High-density lipoprotein^b^		
Poor	Better	
<1.0 mmol/L	≥1.0 mmol/L	

a According to JNC 7 guidelines (Chobanian et al., 2003). ^b^ According to Mayo Clinic guidelines (2021).

### Biophysical Measures

Weight was measured in both pounds (lbs) and kilograms (kg) with a medical grade portable digital scale. Height was measured to the nearest ¼ inch with a wall-mounted tape measure and converted to centimeters. Waist circumference was measured to the nearest ¼ inch about one inch above the umbilicus. Body mass index (BMI) was calculated (kg/m^2^). Using an appropriate cuff size and a medical grade automatic BP monitor (WatchBP Office^©^), BP was measured and recorded 3 times for each arm at 60 s intervals after the participant had been sitting quietly for 5 min. Using blood collected via finger prick method, TC and HDL levels were obtained using the point of care portable Alere Cholestech LDX® Analyzer. See Table 1 for classifications of biophysical measures.

### Survey Measures

The online survey included self-report questions and established scales for the current analysis. CVD risk factors including age, past year smoking status, current diabetes, and current treatment for hypertension required for the calculation of the Framingham 30-year CVD risk scores, and information about exclusion criteria of pre-existing CVD diagnosis and cancer were measured with direct questions (Pencina et al., 2009). Additional CVD risk behaviors were self-reported minutes of physical activity per week, daily servings of fruits and vegetables, and cannabis use. Possible hazardous drinking was indicated by a score greater than 3 on the Audit Alcohol Consumption screen, provided the score did not come only from the frequency of drinking item (Bradley et al., 2007). Indicators of SDOH were captured by self-reports of education status, marital status, sexual orientation, employment status, difficulty living on current income, and frequency of feeling overwhelmed by daily stress.

Gender was measured using the 37-item GRC scale which measures men’s reactions to gender role expectations from 1 (*strongly disagree*) to 6 (*strongly agree*) on four 6-point subscales. Subscale item scores were summed and averaged for a total subscale score of 1 to 6. The Conflict Between Work, Leisure and Family (GRC-CBWF) subscale measures how much difficulty men have balancing demands of work, family, and leisure; the Restrictive Affectionate Behavior Between Men (GRC-RABBM) subscale captures the degree of discomfort or restraint men experience about physical contact, or sharing feelings or care with other men; and the Restrictive Emotionality (GRC-RE) subscale indicates how constrained and fearful men are about expressing emotions to others (O’Neil, 2008). On these subscales, higher scores indicate greater gender role restrictions. The fourth subscale, Success, Power, and Competition (GRC-SPC) assesses personal attitudes about pursuing success through competition and power, with higher scores reflecting greater endorsement (O’Neil, 2015). Over 10 studies, coefficient alphas averaged .80 for GRC-CBWF, .84 for GRC-RE, .84 for GRC-RABBM, and .86 for GRC-SPC (O’Neil, 2015). In this analysis, GRC-CBWF α = .82; GRC-RABBM α = .90; GRC-RE α = .91; and GRC-SPC α = .88

The CLVS-44 scale was used to measure the severity of men’s experiences of physical, psychological, and sexual violence as target and perpetrator, in childhood and adulthood, in the contexts of families, relationships, workplaces, and the community (Scott-Storey (2020). For each of the 44 items, men rated frequency, from 1 (*never*) to 4 (*often*), and degree of distress, from 1 (*not at all*) to 4 (*very*). Frequency and distress scores were summed and averaged for a severity score of 1 to 4 on each item, with higher scores indicating greater severity. Item severity scores were summed and averaged for a total CLVS-44 scale (range 1–4). Internal consistency was .92 in this analysis.

Cardiovascular risk was measured using the Framingham 30-year lipid hard risk scores for coronary death, myocardial infarction, and fatal or non-fatal stroke for men ages 20 to 59 years (Pencina et al., 2009). As per the Framingham guidelines, men with established CVD and/or diagnosis of cancer were excluded. Using the risk calculator (Pencina et al., 2009), 30-year CVD risk scores were calculated using measured mean systolic BP, TC, and HDL, and self-reported age, past year smoking status, presence of diabetes, and hypertension treatment; normal reference scores were calculated using age and sex. Risk scores were classified as: low risk <12%; intermediate risk ≥12% and <40: high risk ≥40% (Pencina et al., 2009).

### Data Analysis

We used IBM^®^ SPSS^®^ Version 28 for analysis. Descriptive statistics were calculated for all variables. For Goal 1, we used paired *t*-tests to compare 30-year CVD risk scores with normal reference scores. After classifying 30-year CVD risk scores into low, intermediate, and high groups as defined by Pencina et al. (2009), we combined the intermediate and high groups to create a dichotomous measure of *low* and *elevated* 30-year CVD risk. These two groups were compared on known CVD risk factors using independent *t* tests and chi-square tests as appropriate. For Goal 2, we compared the *low* and *elevated* 30-year CVD risk groups on CLVS, gender, SDOH, and health behaviors. To achieve Goal 3, we conducted parallel multiple mediation analysis using PROCESS 4.1(Hayes, 2022) to test the hypotheses that CLVS has direct and specific indirect effects through GRC-CBWF, GRC-RABBM, GRC-RE, and GRC-SPC on CVD risk. Known risk factors including age and smoking status were not controlled because they were used to calculate the outcome variable, the Framingham 30-year CVD risk score. Parallel multiple mediation analysis permitted unstandardized estimates of the direct effect CLVS on CVD risk and specific indirect effects through each mediator while controlling for all other mediators as well as CLVS using 10,000 bootstrapped samples and 95% percentile confidence intervals (CI; Hayes, 2022). Direct effects (*c’*) were judged significant when the 95% CI did not contain 0. Indirect effects through each mediator were calculated as the product (*ab*) of the direct effect of CLVS on the mediator (*a*) and the direct effect of the mediator on CVD Risk (*b*) and considered significant when the bootstrapped 95% percentile CI did not contain 0. Because our interest was in the hypothesized specific mechanisms by which CLVS affects CVD Risk, we report specific indirect effects and the direct effect but not the aggregate mechanisms (total effects and total indirect effects) because they have little substantive or theoretical value (Hayes, 2022).

## Results

### The Sample

Of the 244 men who had health assessments, 67 were excluded from the analysis; 24 were not in the eligible age range of 20 to 59 years and 6 reported no CLV. Other exclusions were 20 cases for pre-existing heart disease, 1 for cancer, and 16 for missing data on a variable needed to calculate the 30-year CVD risk score or test the mediation model. Therefore, the total sample for the current analysis was 177. The men had a mean CLV severity score of 1.44 (*SD* = .37) and a mean age of 36.0 (*SD* = 11.17). Slightly more than half (52.5%, *n* = 93) were married or living with a partner; 39.59% (*n* = 70) had dependent children; 90.4% (160) identified as heterosexual; 50.3% (*n* = 89) had completed college or university; 65% (*n* = 115) were employed, and 41.85% (*n* =74) found it difficult or extremely difficult to live on their incomes. Although the 67 participants excluded from the sample were significantly older in years than those included (41.6 *vs* 36.0 years, *t* (89.88) = −2.58, *p =*.011), no significant differences were found between those included and excluded for CLV severity, past year smoking status, systolic BP, TC, HDL, GRC, health behaviors such as cannabis use, exercise or hazardous alcohol use and SDOH such as marital status, employment status, or difficulty living on income.

### Goal 1: Comparison of 30-Year CVD Risk Scores to Reference Normal Scores and Known CVD Risk Factors by 30-Year CVD Risk Groups

For the sample of 177 men with CLVS histories, the 30-year CVD *risk* scores (µ = 14.11, *SE* = .99) were higher (µ difference = 4.64) than Framingham reference *normal* scores (µ = 9.47, *SE* = 0.54), bias-corrected and accelerated (BCa) 95% CI = [3.48, 5.94], *t* (176) = 7.34, *p <*.001, *d* = 0.55. Based on CVD risk scores, we classified 100 men as low CVD risk, 65 as intermediate risk, and 12 as high risk; intermediate and high risk men were merged in an *elevated* 30-year CVD risk group (*n*= 77) for comparison with the *low* 30-year CVD risk group (*n* = 100). In the *low* CVD risk group, 30-year CVD *risk* scores (µ = 4.93, *SE* = 0.28) did not differ significantly from the *normal* scores (µ = 4.64, *SE* = 0.27), BCa 95% CI = [−0.15, 0.79], *t* (99) = 1.33, *p* = .202, *d* = 0.13. However, in the *elevated* CVD risk group, 30-year CVD risk scores (µ = 26.03, *SE* = 1.31) were significantly higher (µ difference = 10.30) than *normal* scores (µ = 15.74, *SE* = 0.71), BCa 95% CI = [8.08, 12.82], *t* (76) = 9.02, *p* < .001, *d* = 1.03.

The 30-year CVD risk factor score differed between low (µ = 4.93, *SD* = 2.82) and elevated (µ = 26.04, *SD* = 11.52) CVD risk groups (*t* (83.02) = −15.71, *p* < .001). As expected, total means scores also differed significantly between *low* and *elevated* risk groups for CVD risk factors used to calculate the 30-year CVD risk scores (see Table 2). Men in the elevated risk group had significantly higher weights and BMIs and were more likely to have a waist greater than 40 inches and a family history of early onset heart disease.

**Table 2.** Descriptive Comparative Profile of Cardiovascular (CVD) Risk Factors by Low and Elevated 30-year CVD Risk^a^ Groups (N = 177)

Cardiovascular risk factors	Full sample (*N* = 177)^b^*M* (*SD*)	Low CVD risk (*n* = 100)^b^*M* (*SD*)	Elevated CVD risk (*n* = 77)^b^*M* (*SD*)	Test of differences
Framingham 30-year Lipid Hard CVD Risk Score	14.11 (13.11)	4.93 (2.81)	26.04 (11.53)	*t* (83.02) = −15.71 *p ≤*.001
Age	35.97 (11.17)	28.21 (5.73)	46.05 (8.01)	*t* (131.94) = −16.56 *p ≤*.001
Weight (lbs)	199.20 (47.70)	191.01 (40.49)	209.83 (54.14)	*t* (175) = −2.65 *p* =.009
Body mass index	28.24 (6.18)	26.82 (5.17)	30.08 (6.90)	*t* (175) = −3.59 *p* < .001
Total cholesterol (mmol/L)	4.55 (1.00)	4.26 (0.76)	4.91 (1.16)	*t* (124.56) = −4.29 *p* <.001
High density lipoprotein (mmol/L)	1.18 (0.37)	1.25 (0.39)	1.08 (0.33)	*t* (173.74) = 3.16 *p* = .002
Mean systolic BP	128.24 (11.93)	126.56 (10.98)	130.41 (12.80)	*t* (175) = −2.15 *p* = .033
Mean diastolic BP	79.95 (9.70)	77.40 (8.58)	83.28 (10.10)	*t* (148.67) = −4.10 *p* < .001
	*N* (%)	*n* (%)	*n* (%)	
Diabetes				
Yes	9 (5.1)	1 (1.0)	8 (10.4)	χ^2^ (1) = 7.95 *p* = .005
No	168 (94.9)	99 (99.0)	69 (89.6	
Smoked in past 12 months				
Yes	56 (31.6)	24 (24.0)	32 (41.6)	χ^2^ (1) = 6.20 *p* = .013
No	121 (72.1)	76 (76.0)	45 (58.4)	
Family history of early heart disease				
Yes	34 (19.2)	10 (10.0)	24 (31.2)	χ^2^ (2) = 12.94 *p* = .002
No	112 (63.3)	71 (72.0)	40 (51.9)	
Don’t know	31 (17.5)	18 (18.0)	13 (16.9)	
Body mass index				
<25	54 (30.5)	41 (41.0)	13 (16.9)	χ^2^ (1) = 11.94 *p* <.001
≥25 (overweight or obese)	123 (69.5)	59 (59.0)	64 (83.1)	
Waist circumference	(*n* =176)			
Healthy waist (<40 inches)	116 (65.9)	79 (79.0)	37 (48.7)	χ^2^ (1) = 17.66 *p* < .001
High waist circumference (≥40 inches)	60 (33.9)	21 (21.0)	39 (51.3)	

*Note.* CVD = cardiovascular disease; BP = blood pressure.

a CVD Risk refers to Framingham 30-year Hard Lipid Cardiovascular Risk classified as *Low Risk* < 12% and *Elevated Risk =*> 12% (includes intermediate and high risk scores). ^b^ Unless otherwise specified.

### Goal 2: Descriptive Comparative Profile of CLVS, GRC, SDOH, and Health Behaviors by 30-year CVD Risk Groups

No significant differences in CLVS total mean scores were found between low and elevated 30-year CVD risk groups (see Table 3). GRC differed significantly on three GRC sub-scales. Compared the elevated CVD risk group, men in the low CVD risk group had higher mean scores for the GRC-SPC subscale indicating a stronger endorsement of the masculine norm of pursuing success through competition and power. The low CVD risk group also had higher scores than the elevated risk group on the GRC-CBWF subscale suggesting greater conflict in balancing work, school, and family relations. In contrast, those in the elevated CVD risk group had higher scores for GRC-RABBM suggesting that they had more difficulty touching or expressing care and concern for other men than those in the low CVD risk group. No statistically significant differences between CVD risk groups were found for the GRC-RE subscale or for other SDOHs, specifically, sexual orientation, marital status, education, employment, difficulty living on income, or weekly stress. With respect to health behaviors, the elevated 30-year CVD risk group was significantly less likely than the low CVD risk group to a) eat 5 or more servings of fruits and vegetables per day, and b) be hazardous drinkers. No significant differences were found between groups for cannabis use and weekly hours of exercise.

**Table 3.** Descriptive Profile of Cumulative Lifetime Violence Severity, Gender, Social Determinants of Health, and Health Behaviors by CVD Risk Groups^a^ (*N* = 177).

Variable	Full sample (*N* = 177) *M* (*SD*)	*Low* CVD risk (*n* = 100) *M* (*SD*)	*Elevated* CVD risk (*n* = 77) *M* (*SD*)	Independent *t-*tests
CLVS-44^b^ total	1.44 (0.37)	1.41 (0.35)	1.47 (0.38)	*t* (175) = −1.025, *p* = .153
Gender role conflict				
Conflict between work, and family	3.36 (1.15)	3.51 (1.19)	3.17 (1.06)	*t* (175) = −1.97, *p* = .05*
Restrictive affectionate behavior between men	2.89 (1.16)	2.69 (1.02)	3.15 (1.26)	*t* (144.47) = −2.57, *p* = .011*
Restrictive emotionality	3.24 (1.22)	3.28 (1.10)	3.18 (1.36)	*t* (143.37) = 0.511, *p* =.610
Success, power, competition	3.36 (0.99)	3.57 (1.04)	3.08 (0.87)	*t* (175) = 3.31, *p* = .001*
	*n* (%)	*n* (%)	*n* (%)	χ^2^
Sexual orientation				
Heterosexual	160 (90.4)	87 (87.0)	73 (94.8)	χ^2^(1) = 3.05, *p =* .081
Gay, bisexual, other	17 (9.6)	13 (13.0)	4 (5.2)	
Marital status	(*n* = 176)	(*n* = 99)		
Married/living together	93 (52.5)	48 (48.5)	45 (58.4)	χ^2^(1) = 1.72, *p =* .224
Single, divorced, separated, widowed	88 (46.9)	51 (51.5)	32 (41.6)	
Education				
University or college completed	89 (50.3)	53 (53)	36 (46.8)	χ^2^(1) = 0.68, *p =* .450
Less than university or college completion	88 (49.7)	47 (47)	41 (53.2)	
Employment				
Employed	115 (65.0)	68 (68.0)	47 (61.0)	χ^2^(1) = 0.93, *p =* .345
Unemployed	62 (35.0)	32 (32.0)	30 (39.0)	
Difficulty living on Income	(*n* = 176)		(*n* = 76)	
Not at all to a little difficult	102 (58.0)	60 (60.0)	42 (55.3)	χ^2^(1) = 0.40, *p =* .541
Difficult to extremely difficult	74 (42.0)	40 (40.0)	34 (44.7)	
Overwhelmed by stress in a typical week				
Often, most of the time	54 (30.5)	32 (32.0)	22 (28.6)	χ^2^(1) = 0.24, *p =* .742
Never, seldom, a few times	123 (69.5)	68 (68.0)	55 (71.4)	
Physical activity weekly				
150 min or more	75 (42.4)	46 (46.0)	29 (37.7)	χ^2^(1) = 1.23, *p =* .286
<150 min	102 (57.6)	54 (54.0)	47 (47.1)	
Daily servings fruits and vegetables	(*n* = 176)	(*n* =99)		
<5	142 (80.2)	71 (71.7)	71 (92.2)	χ^2^(1) = 11.67, *p* < .001 *
5 or more	34 (19.2)	28 (28.3)	6 (7.8)	
Possible hazardous drinking	(*n* = 176)	(*n* = 99)		
Yes	101 (57.1)	65 (65.7)	36 (46.8)	χ^2^(1) = 6.33, *p* < .014*
No	75 (42.4)	34 (34.3)	41 (53.2)	
Cannabis use				
Daily	40 (22.6)	25 (25.0)	15 (19.5)	χ^2^(1) = 0.76, *p* = .469
Never, a few times, monthly, weekly	137 (77.4)	75 (75.0)	62 (80.5)	

*Note.* CVD = cardiovascular disease; CLVS = cumulative lifetime violence severity.

a CVD Risk refers to Framingham 30-year Hard Lipid Cardiovascular Risk classified as *Low Risk* < 12% and *Elevated Risk* > 11% (includes intermediate and high scores). ^b^ CLVS-44 refers to the 44-item Cumulative Lifetime Violence Severity Scale.

* Significant at .05 or less.

### Goal 3: Testing the Hypothesis That CLVS has Both Direct and Specific Indirect Effects Through GRC on 30-Year CVD Risk

**Figure 1.**
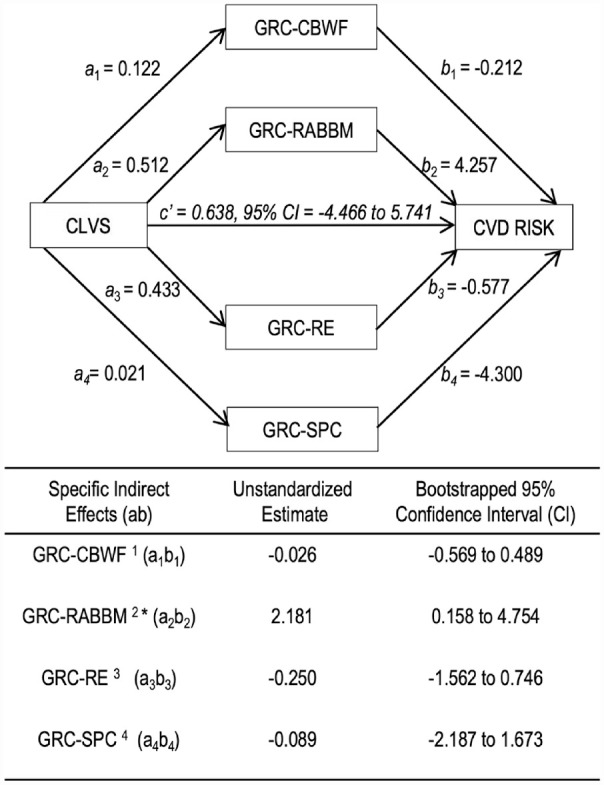
Parallel Multiple Mediation Model Depicting Unstandardized Direct and Specific Indirect Effects of Cumulative Lifetime Violence Severity (CLVS) Through Gender Role Conflict (GRC) Subscales on Cardiovascular (CVD) Risk

As depicted in Figure 1, CLVS did not directly influence 30-year CVD risk (*c*’ = 0.638, *p* = .806, 95% CI = [−4.466, 5.741]. CLVS significantly affected 30-year CVD risk indirectly through GRC-RABBM (*a_2_b_2_ =* 2.181, 95% bootstrapped CI = [0.158, 4.754)]. Men who experienced greater CLVS reported higher GRC in physical contact and expression of feelings toward men (*a_2_* = 0.512), and this higher GRC-RABBM was associated with higher 30-year CVD risk (*b_2_* = 4.257). Independent of this mechanism, no other specific indirect relationships were found between CLVS and 30-year CVD risk.

## Discussion

Our empirical findings offer new insights regarding the mechanisms by which violence as a chronic toxic stressor is associated with increased risk for CVD among men (Kivimäki & Steptoe, 2018; Scott-Storey, 2013). Critically, we determined that in a community sample of 177 men ages 20 to 59 years who reported *cumulative* lifetime violence as target and/or perpetrator, 30-year CVD risk scores were 1.5 times higher than the reference normal values for men of the same ages, a finding that highlights the importance of CLVS as a SDOH that may increase CVD risk among men. Striking is the finding that in contrast to the *low* 30-year CVD risk group (*n* =100) where CVD risk scores were not significantly different from the expected normal, the *elevated* CVD risk group (*n* = 77) had CVD risk scores an average of 1.7 times higher than reference normal values. Important context for these findings is that 20 other men in the sample who were between 20 and 59 years old were excluded from calculation of 30-year CVD risk because of *pre-existing* heart disease; that is, they were ineligible for risk assessment because they had heart disease. Therefore of 197 men ages 20 to 59 years old with histories of CLV, almost *half* (*n* = 97) already have heart disease or are at elevated risk for CVD. Because the relationship between CLV and CVD risk has not been previously studied, these results provide a benchmark for future study of this relationship in community samples of men reporting CLV.

Importantly, men with fewer than 2 risk factors at age 50 are less likely to develop CVD and to live longer than those with 2 or more risk factors (Lloyd-Jones et al., 2006). The mean age of men in the *elevated* risk group was 46.05 (*SD* 8.01) years, suggesting that, for many, reduction of CVD risk factors to improve outcomes is possible. Violence affects CVD not only through allostatic overload but also through coping strategies known to contribute to CVD; therefore, TVI prevention strategies that move beyond a narrow focus of behavioral change to validate and address the impacts of past and ongoing violence (Wathen et al., 2021) are critical for men with CLV histories. In the *elevated risk* group, 41.6% of men were smokers, more than double the rate for Canadian men overall (17.3%) (Statistics Canada, 2020), 83.1% were overweight or obese as compared with 69.4% of Canadian men in general (Statistics Canada, 2019), and 92.2% consumed fewer than 5 servings of fruits and vegetables associated with lower CVD mortality (Wang et al., 2021). A TVI approach that builds on men’s strengths, and enhances safety and choice (Ponic et al., 2016) may help men in both *elevated* and *low risk* groups modify their CVD risk. In this sample, a greater proportion of men in the *low* CVD than the *elevated* CVD risk group were hazardous drinkers. Possibly the younger mean age of the *low* CVD risk group (28.21) accounts for different patterns of alcohol use; hazardous drinking may be a more acceptable coping strategy for violence-related stress among younger men. Given the established association between hazardous drinking and CVD morbidity and mortality (Arora et al., 2022), this finding highlights a TVI pathway for supporting increased control over longer term CVD outcomes that might be acceptable to some younger men with CLV.

Despite the robust existing evidence that other SDOH such as socio-economic factors are associated with CVD (Schultz et al.), gender was the only SDOH with a significant association with CVD risk. Limited research has focused on the health consequences of trauma, such as violence, in adults by gender (Lewis-O’Connor et al. 2019). The findings that men in the *low* 30-year CVD risk group had significantly higher GRC-SPC scores than men in the *elevated* CVD risk group suggest that higher success, power, and control scores may be protective. Unlike other GRC subscales that measure negative outcomes associated with traditional gender role socialization, most SPC items assess masculine norms/ideology in terms of personal attitudes and values related to success through competition and power (O’Neil, 2015). Thus, strong beliefs that masculinity demands being in charge, winning, and being smarter and stronger than others may shield some men from feeling at risk or victimized by violence, limit distress, and/or mitigate biophysical responses to experiences of violence that might otherwise increase CVD risk. These strong beliefs may be a resource for men who choose to take control of lifestyle factors salient in the development of CVD.

Our comparative analysis also showed that the *low* 30-year CVD risk group had higher GRC in balancing work, school, leisure, and family. These men were significantly younger than those in the *elevated* risk group (mean age 28.21 vs. 46.05 years) indicating that differences in GRC-CBWF may be associated with different profiles of family responsibilities and career demands. Men in the *low* CVD risk group may have growing families and more career development pressures than men in the *elevated* CVD risk group whose children may be older and whose careers may be more established. This knowledge calls attention to the importance of CVD health promotion strategies to help younger men with CLV experiences strengthen their capacity to balance the demands of work and family more effectively. We also found that men in the *elevated* CVD risk group had higher scores on GRC-RABBM than those in the *low* CVD risk group. This subscale captures how much men who have been socialized to believe that men do not express care and concern for other men endure conflict (e.g., fear, hesitancy, discomfort) related to showing feelings for or having physical contact with other men (O’Neil, 2015). Such GRC in men with CLV may be an additional stressor that increases their vulnerability for CVD over time, particularly among men whose occupations require physical contact or social interaction with men such as first responders, those in policing or the military.

Our findings are some of the first to illuminate mechanisms by which gender is associated with 30-year CVD risk in men with CLV experience. Although our hypothesis that CLVS has a direct effect on CVD risk was not supported, we found that the relationship between CLVS and CVD risk was significantly mediated by GRC-RABBM in a model controlling for the effects of other mediators (GRC-SPC, GRC-RE, GRC-CBWF). To our knowledge, this is the first analysis illuminating the mechanism by which gender, specifically GRC-RABBM, affects 30-year CVD risk scores. Perhaps, for some men, the chronic and recurring violence early in life and into adulthood primes them to fear close physical or emotional contact with other men as a violation of dominant norms causing excessive distress that over time may contribute to an increase of CVD risk. Importantly, gender role socialization is not homogeneous across cultures or social groups and these findings may not be applicable to all Canadian men. But recognition of the way GRC related to the norm of avoidance of physical and emotional closeness with men may negatively affect cardiovascular health provides an opportunity to consider how social and public policy might be used to modify this dominant belief among men.

### Limitations

The study has several limitations. The sample is relatively small and consists primarily of White men living in eastern Canada. Further testing of this model in larger, more diverse samples is required. The men in this study were mostly heterosexual and findings related to GRC may not hold for a sample that includes a larger proportion of gay, bisexual and/or transgender men. In addition, the CLVS-44 is a new measure developed for the *MVGHS* survey from which these data are drawn (Scott-Storey et al., 2020). However, factor structure was confirmed in a second ongoing study with a sample of men with CLV located in all Canadian provinces.

### Conclusion

Our key findings offer vital new evidence that gender and severity of CLV may affect CVD risk in men. In general, men reporting CLV histories were found to have significantly higher 30-year CVD risk scores than expected normal scores for men of the same age, a pattern that was more remarkable among men in the *elevated* CVD risk group. Also, novel is our finding of the specific significant indirect effect of GRC, specifically restrictive affectionate behavior between men (GRC-RABBM) as a mediator between CLVS and CVD risk. To our knowledge, no previous research has identified a link between CLV and CVD risk through gender. These results reinforce the critical role of chronic toxic stress, particularly from CLV across the lifespan but also from GRC, in influencing CVD risk (Kivimäki & Steptoe, 2018; Scott-Storey, 2013). Together these outcomes draw attention to the need for TVI policy and interventions that recognize that social determinants may intersect to negatively affect men’s cardiac health.
